# Unraveling the molecular determinants of the anti-phagocytic protein cloak of plague bacteria

**DOI:** 10.1371/journal.ppat.1010447

**Published:** 2022-03-31

**Authors:** Daniel T. Peters, Antonio Reifs, Alvaro Alonso-Caballero, Azzeldin Madkour, Helen Waller, Brendan Kenny, Raul Perez-Jimenez, Jeremy H. Lakey

**Affiliations:** 1 Biosciences Institute, Newcastle University, Newcastle upon Tyne, United Kingdom; 2 CIC nanoGUNE BRTA, San Sebastian, Spain; 3 Ikerbasque Foundation for Science, Bilbao, Spain; University of Pennsylvania, UNITED STATES

## Abstract

The pathogenic bacterium *Yersina pestis* is protected from macrophage engulfment by a capsule like antigen, F1, formed of long polymers of the monomer protein, Caf1. However, despite the importance of this pathogen, the mechanism of protection was not understood. Here we demonstrate how F1 protects the bacteria from phagocytosis. First, we show that *Escherichia coli* expressing F1 showed greatly reduced adherence to macrophages. Furthermore, the few cells that did adhere remained on the macrophage surface and were not engulfed. We then inserted, by mutation, an “RGDS” integrin binding motif into Caf1. This did not change the number of cells adhering to macrophages but increased the fraction of adherent cells that were engulfed. Therefore, F1 protects in two separate ways, reducing cell adhesion, possibly by acting as a polymer brush, and hiding innate receptor binding sites needed for engulfment. F1 is very robust and we show that *E*. *coli* expressing weakened mutant polymers are engulfed like the RGDS mutant. This suggests that innate attachment sites on the native cell surface are exposed if F1 is weakened. Single-molecule force spectroscopy (SMFS) experiments revealed that wild-type F1 displays a very high mechanical stability of 400 pN. However, the mechanical resistance of the destabilised mutants, that were fully engulfed, was only 20% weaker. By only marginally exceeding the mechanical force applied to the Caf1 polymer during phagocytosis it may be that the exceptional tensile strength evolved to resist the forces applied at this stage of engulfment.

## Author summary

Macrophages, a type of white blood cell, form an important element of our immune defence. They interrogate other cells’ surfaces for molecular clues and ingest those presenting a threat in a process known as phagocytosis. Not surprisingly, pathogenic bacteria have developed ways to evade this fate. The plague bacterium, *Yersinia pestis*, produces the long polymeric F1 coat protein which enables it to avoid ingestion, but the mechanism was unclear. We show that equipping *Escherichia coli* cells with an F1 coat protected them from phagocytosis by two separate mechanisms, reducing contact with the macrophage surface and hiding the signals that tell the macrophages they are targets. F1 is also a very stable protein polymer and using single molecule force spectroscopy we showed it also has a very high resistance to pulling forces. Surprisingly, mutations which reduced this by only 20% caused adherent bacteria to be fully ingested, indicating that cells are subject to significant forces prior to recognition and ingestion. Thus, F1 has evolved three notable properties (i) physical; creation of a hydrated polymer brush to inhibit surface interactions, (ii) chemical; absence of molecular recognition clues needed for engulfment and (iii) mechanical; strength that maintains the camouflage layer during surface stretching.

## Introduction

Macrophages recognise their targets through a wide variety of cell surface receptors [1–3], attacking foreign bodies such as bacterial cells by recognising either (i) particular molecules, present in these organisms but not the host, called pathogen associated molecular patterns (PAMPs) or (ii) opsonising molecules such as complement. Once recognised, the macrophage engages the actin cytoskeleton to draw the prey into the newly formed phagocytic cup, where it is engulfed by the macrophage and degraded in a specialised organelle called the phagosome. Different biophysical approaches have shown that macrophage filopodia exert forces over a broad pN-nN range as they attach to microparticles and draw them towards the cell body for engulfment [4–6]. Receptor binding has been shown to be essential for phagocytosis [2,3,6] but the biomechanics involved are not fully understood [7,8].

Bacterial pathogens can avoid destruction via a diverse array of strategies including subverting the phagosome to enable intracellular expansion within the macrophage [9] or through masking their distinctive surface by hiding behind a capsule to avoid detection by cells of the immune system [10]. *Yersinia pestis*, the causative agent of the plague, uses both strategies [11]. *Yersinia* do not express a protective capsule at the low temperature of their flea vector and, immediately following infection of the host via a flea bite, they are readily engulfed by neutrophils and macrophages. Whilst within the phagosome they inhibit the destructive pathway and expand in numbers whilst at the same time activating operons such as *yop* and *caf*. Following escape from the initial host-macrophage these proteins provide protection from further engulfment and allow systemic infection to proceed [9,11–13].

The Yop (*Yersinia* outer membrane protein) system injects phagocytosis inhibiting proteins into macrophages via a type III secretion system (T3SS) [14–16] whilst Caf1 (capsular antigen fraction 1) enables passive phagocytosis resistance by cloaking *Y*. *pestis* in a gel-like protein coat termed F1 [17]. Caf1 is a 15 kDa protein that assembles into the long, non-covalent, extracellular polymer (F1), via the chaperone-usher (CU) pathway [18]. This surrounds the bacterium [17] and enables *Y*. *pestis* cells to avoid macrophage engulfment [17,19,20]. Crucially, purified F1 does not inhibit the phagocytosis of co-administered yeast cells and therefore does not inhibit the phagocytic activity of macrophages [17]. Instead F1 prevents the association of *Y*. *pestis* bacteria with the macrophages, suggesting that it is anti-adhesive [17]. *In vitro* studies have shown that the “non-stick” phenotype of F1 extends to cell types other than macrophages, with a wide range of cells adhering very poorly to F1 treated surfaces in culture [21].

Here, we investigated the molecular determinants of F1’s anti-phagocytic activity. We first determined that recombinant *E*. *coli* coated with F1 evaded engulfment by macrophages through two effects. Firstly, compared to controls, far fewer F1 coated bacteria attached to the surface of the macrophage, and secondly, of those that did attach, very few were phagocytosed. We then showed that insertion of an integrin binding motif into F1 did not alter the number of *E*. *coli* attached to the macrophage but did enable engulfment of the attached bacteria. Next, we investigated the effect of single amino acid substitutions which reduce F1 polymer stability and find that they also allow engulfment of adhered bacteria. Single-molecule force spectroscopy (SMFS) then revealed not only that F1 has an exceptionally high tensile strength but also that the destabilising mutations cause a drop in strength of only ~20%. This suggests that the wild type strength is only just sufficient to resist the forces applied during the phagocytosis of adhered bacteria. This is in agreement with the general theory of protein marginal stability whereby proteins evolve to just exceed the stability required for their function [22]. The combined results suggest that three key properties of F1 –its low non- specific affinity for cells, its absence of ligands for macrophage receptors and its exceptional mechanostability–have co-evolved to generate the anti-phagocytic property of the protein, contributing significantly to the virulence of the plague pathogen. Furthermore, since there is still much to be learned about the biomechanics of engulfment, the data presented here offer a new insight into the pathogen-macrophage interface.

## Results

### Expression of *caf1* in *E*. *coli* confers anti-phagocytic ability

**Fig 1:**
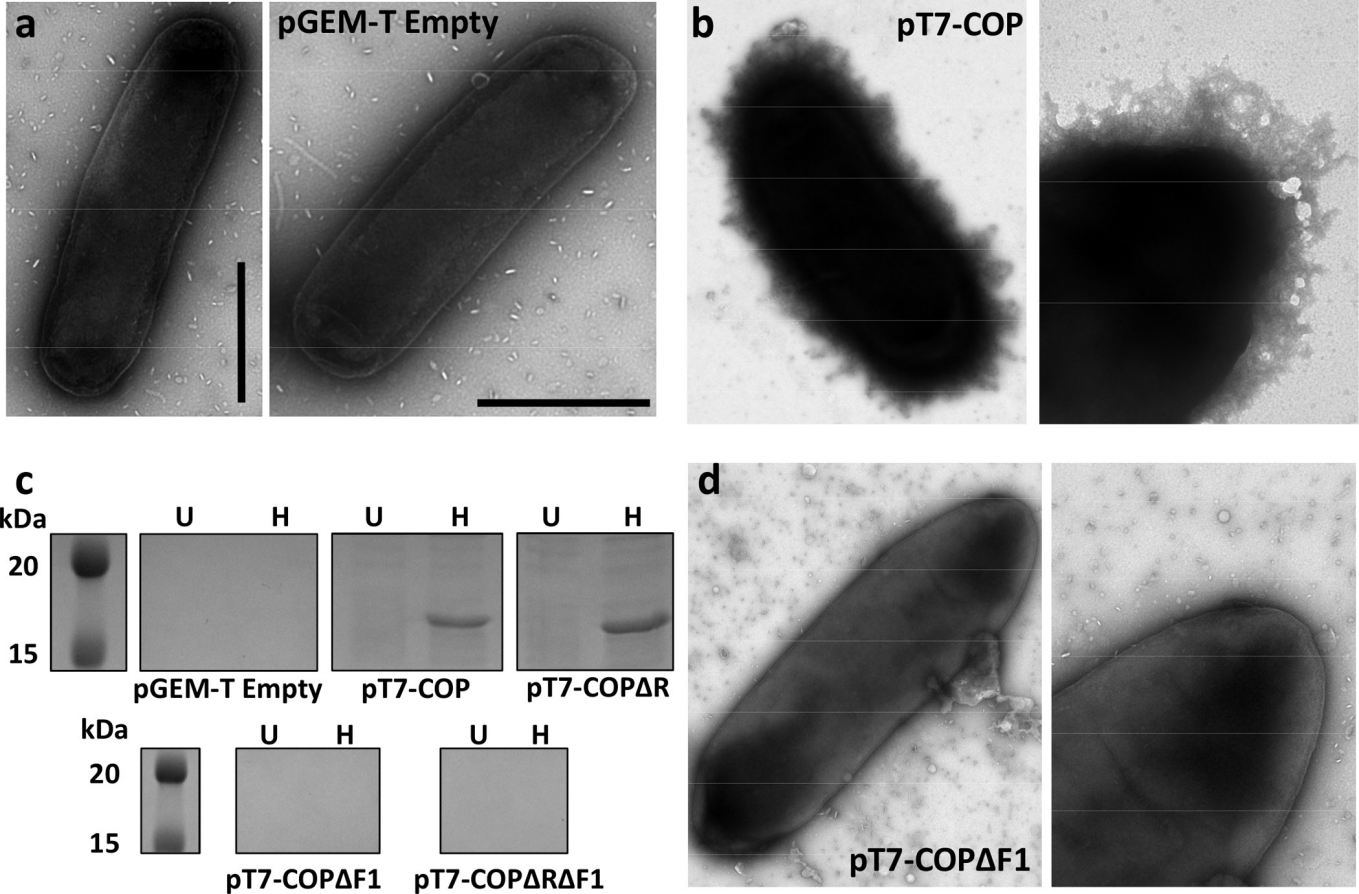
Expression of *caf1* in *E*. *coli* results in capsule formation. Transmission electron micrographs of *E*. *coli* transformed with either empty vector (pGEM-T) (**a**) or pT7-COP (**b**). (**c**) SDS-PAGE analysis of the extracellular fraction (comprising flocculent and supernatant) of cultures of *E*. *coli* transformed with the indicated plasmids and grown for 22 h at 35°C. Samples were incubated at either room temperature (unheated, U) or 100°C (heated, H) for 5 min prior to loading on the gel. (**d**) Transmission electron micrographs of *E*. *coli* transformed with pT7-COPΔF1. Scale bar represents 500 nm.

*Y*. *pestis* produces a Caf1 coat upon transfer from a flea vector to a warm-blooded host, enabling it to evade phagocytosis [17]. To determine if heterologous expression of *caf1* provides *E*. *coli* cells with a protective coat, BL21(DE3) cells were transformed with either empty pGEM-T vector or the pT7-COP plasmid, in which pGEM-T contains the entire *caf1* operon (*caf1R*, *caf1M*, *caf1A* and *caf1*). These cells were grown for 22 h at 35°C to induce *caf1* expression, and then imaged using transmission electron microscopy (TEM). Compared to the cells transformed with the empty vector, cells expressing *caf1* appeared to be surrounded by an amorphous gel-like coat (**Fig 1A and 1B**). The images are similar to those of *Y*. *pestis* bacteria expressing *caf1* [23]. Therefore, heterologous expression of the *caf1* operon results in the same morphological phenotype as in the natural system, providing *E*. *coli* with a Caf1 capsule.

**Fig 2:**
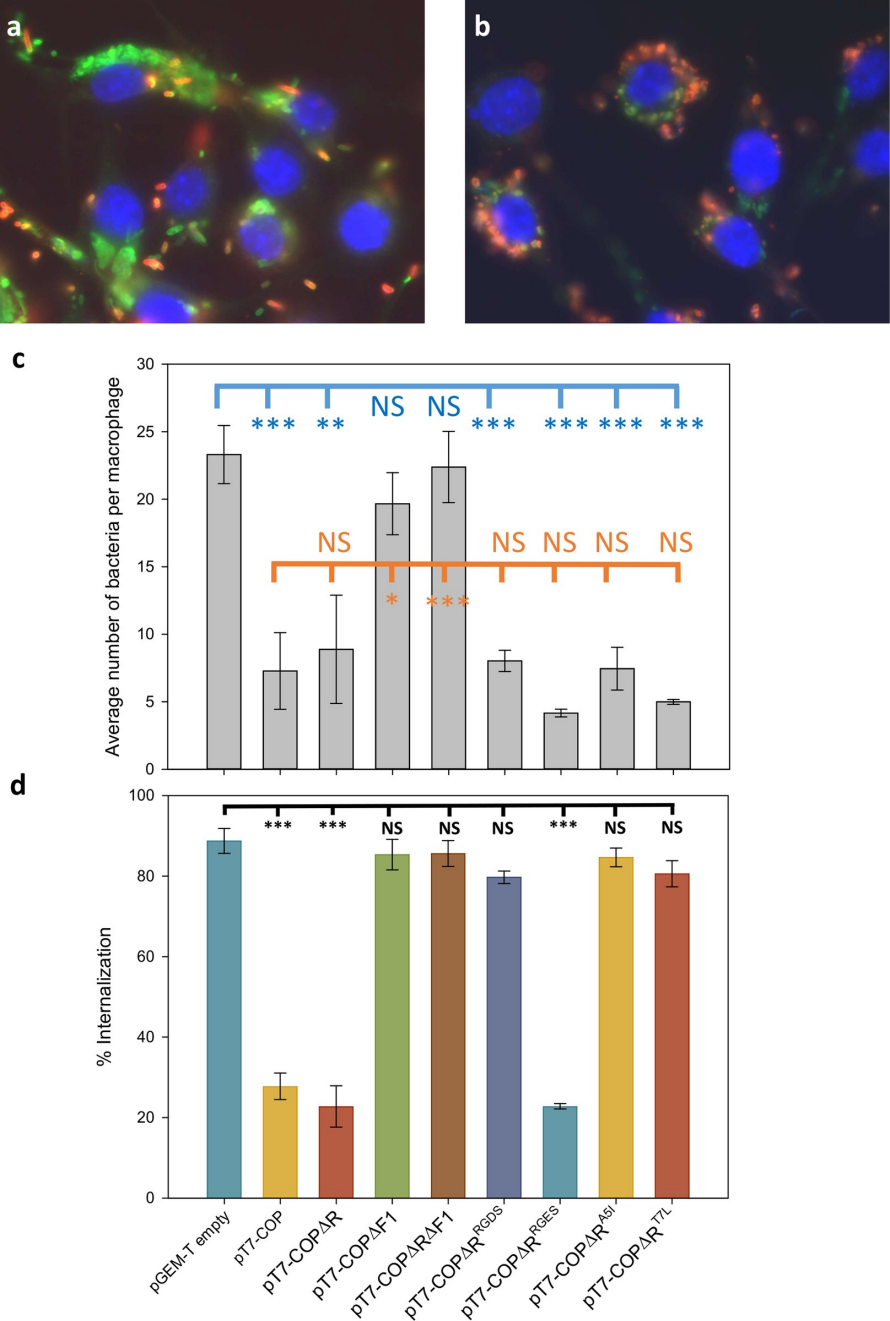
Expression of *caf1* protects *E*. *coli* from phagocytosis. BL21(DE3) *E*. *coli* cells transformed with the indicated plasmids were grown for 18 h and used to infect J774.A1 macrophages for 2 h. Bacteria external to the macrophages were labelled in red and all bacteria labelled green, with macrophage nuclei stained blue. Merged microscopy images are shown for bacteria transformed with pT7-COPΔRΔF1 (**a**) and pT7-COPΔR (**b)** in which external bacteria stained both green and red appear yellow to orange. Images were taken with a Zeiss Axioskop Epifluorescence microscope with a 100x oil objective. (**c**) Average number of bacterial cells counted per macrophage. Error bars represent the standard deviation from three biological replicates. Bars correspond to the same samples as shown in (**d**). Significant differences were detected by one way ANOVA followed by a Holm-Sidak post-hoc test (**d**) Percentage of bacterial cells internalised by J774.A1 macrophages. Fifty macrophages were randomly chosen to calculate internalisation, with the identity of the sample unknown to the experimenter. Percentages were calculated as the number of green bacteria minus red bacteria (= internalized organisms) divided by the number of green bacteria (= total cell associated bacteria). Error bars represent the S.E.M of three independent biological replicates. Data were analysed by one way ANOVA with a Holm-Sidak post-hoc test. * = p < 0.05, ** = p, 0.01, *** = p < 0.005, NS = not significant.

Next, to determine whether this capsule possessed the same anti-phagocytic properties as those described for *Y*. *pestis*, we transformed *E*. *coli* with pGEM-T, pT7-COP and pT7-COPΔF1, where *caf1* translation is prematurely terminated through the introduction of a stop codon. Production of Caf1 polymers produces a flocculent layer which can be seen above the cell pellet after centrifugation [24]. Cells containing the pT7-COP plasmid produced a flocculent layer (**S1A Fig**), and Caf1 polymers could be detected in the extracellular fraction of the culture using SDS-PAGE (**Fig 1C**), whereas cells containing pGEM-T or pT7-COPΔF1 showed no detectable flocculent layer or Caf1 protein. Additionally, an F1 coat could not be observed by TEM on pT7-COPΔF1 transformed cells (**Fig 1D**). These cell cultures were used to infect J774.A1 macrophages at a multiplicity of infection of 100:1 bacteria:macrophage before fixing. The number of *E*. *coli* engulfed by the macrophages was then determined by a previously described immunofluorescence assay [25] where extracellular bacteria are labelled red and all bacteria (intra- and extracellular) are labelled green. Macrophages were then examined by fluorescence microscopy, and the number of green and red stained bacteria calculated to determine the percentage of engulfed cells (**Figs 2** and **S2**). This was calculated as the number of green bacteria minus red bacteria (= internalized organisms) divided by the number of green bacteria (= total cell associated bacteria). The comparisons were made using three biological replicates in a blind assay.

These data gave three important results. Firstly, that Caf1’s anti-phagocytic activity can be easily transferred to *E*. *coli*. Secondly, that the total number of cells associated with the macrophages is reduced by about 70% in F1 coated compared to uncoated bacteria (**Fig 2C**) and thirdly that even macrophage-bound bacteria were protected from engulfment when *caf1* was expressed (**Fig 2D**).

The Caf1R protein regulates *caf1* expression as part of a complex thermosensitive system that responds to host body temperature [26,27]. To ensure reproducible Caf1 levels we simplified *caf1* expression in our assays by deleting *caf1R*, and so used only basal T7 expression from pT7-COPΔR. The cells transformed with this plasmid displayed the expected F1 coat when observed by TEM (**S3A Fig**). The macrophage assay was thus repeated using pT7-COPΔR, with pT7-COPΔRΔF1 as a control. Expression of *caf1* from pT7-COPΔR allowed the *E*. *coli* to avoid phagocytosis, with a drop in internalisation similar to that seen in the presence of Caf1R, whereas prevention of *caf1* translation again reversed this effect and allowed the phagocytosis of the bacteria (**Fig 2D**). Therefore, the ability to evade phagocytosis by macrophages can be conferred to *E*. *coli* in a *caf1* dependent, *caf1R* independent, manner.

### Addition of a cell binding motif reverses anti-phagocytic activity of Caf1

F1 has previously been shown to possess “non-stick” properties, with mammalian cells in 2D cell culture adhering very poorly to F1 coated surfaces. This phenotype was reversed through the addition of the integrin binding motif, RGDS, which then facilitated cell attachment to the F1 surface [21]. To investigate whether this “non-stick” property of F1 has a role in its anti-phagocytic activity, *E*. *coli* cells were transformed with pT7-COPΔR^RGDS^ in which Caf1 contains the RGDS integrin binding motif inserted within loop 5 of the protein (between residues N106 and D111 in the mature sequence), or with pT7-COPΔR^RGES^, which has the RGES motif at the same site. The RGES motif differs from the RGDS motif by a single -CH_2_- group but does not support cell adhesion. Loop 5 was chosen as it is exposed on the surface of Caf1 and the insertion does not reduce purification yields [21,28]. When the bacteria containing these plasmids were grown for 22 h at 35°C, the cells were seen by TEM to be surrounded by a capsule (**S3 Fig)**. Additionally, a flocculent layer was visible above a centrifuged cell pellet (**S1A Fig**), and F1 polymers could be detected by SDS-PAGE (**S1B Fig**).

The transformed cells were then used to infect cultures of macrophages in the phagocytosis assay. The results revealed that adding the integrin binding motif to Caf1 abolished the anti-phagocytic phenotype, allowing engulfment of the majority of the bacteria adhered to the macrophages (**Fig 2D**). The control F1^RGES^ retains the full anti-phagocytic effect of F1, showing that engulfment of the RGDS mutant occurs through specific integrin recognition. Interestingly, F1^WT^, F1^RGDS^ and F1^RGES^ variants all reduced the total number of bacteria attached to the macrophages, with the latter binding least, (**Fig 2C**) showing that the low non-specific adherence is unaffected by integrin binding. The results demonstrate that low non-specific cell adherence and lack of specific ligands for phagocytic cell receptors contribute separately to F1’s protective phenotype.

### F1’s exceptional stability is essential for protection from phagocytosis

Caf1 subunits assemble into F1 polymers non-covalently through the process of donor strand complementation, where the N-terminal β-strand of one Caf1 subunit completes the Ig-like fold of the next subunit in the polymer [29]. This results in polymers with exceptionally high chemical [30], mechanical [31] and thermo-stability [30]. We wanted to investigate the role of polymer stability in F1 function, and so examined the effect of two single amino acid substitutions in the donor β-strand (Ala-5 to Ile [A5I] and Thr-7 to Leu [T7L]). These proteins form normal length polymers but with a thermostability lowered by ~7°C [32,33].

The macrophage assays were repeated using *E*. *coli* cells transformed with the pT7-COPΔR^A5I^ and pT7-COPΔR^T7L^ plasmids. Expression of the Caf1^A5I^ and Caf1^T7L^ mutants provided *E*. *coli* with a F1 capsule visible by TEM (**S3 Fig**), that had a similar appearance to the wild-type protein. Both the Caf1^A5I^ and Caf1^T7L^ mutants resulted in a flocculent layer (**S1 Fig**) and in Caf1 polymers detectable by SDS-PAGE (**S1 Fig**).

In phagocytosis assays, bacteria expressing these mutants still bound poorly to macrophages (**Fig 2C**) but were engulfed at high levels (**Fig 2D**), similarly to cells expressing the RGDS mutant, demonstrating that these single amino acid substitutions abrogate F1’s ability to prevent the phagocytosis of bound cells. This result was surprising, as the experiments were conducted at 37°C, far below the reduced melting temperature of the proteins (~80°C [33]).

### Lower stability Caf1 polymers are not recognised by macrophages

To determine whether the loss of protection by these mutant F1 polymers was caused by an increase in their affinity for macrophage receptors, we performed a phagocytosis assay using F1 coated polystyrene beads, rather than bacteria. As these beads lack the macrophage recognition sites present on the bacterial outer membrane, any increase in the ability of macrophages to engulf beads coated with the mutant proteins over beads coated with the wild-type protein will be caused by increases in the ability of the macrophages to recognise and bind to the proteins.

**Fig 3:**
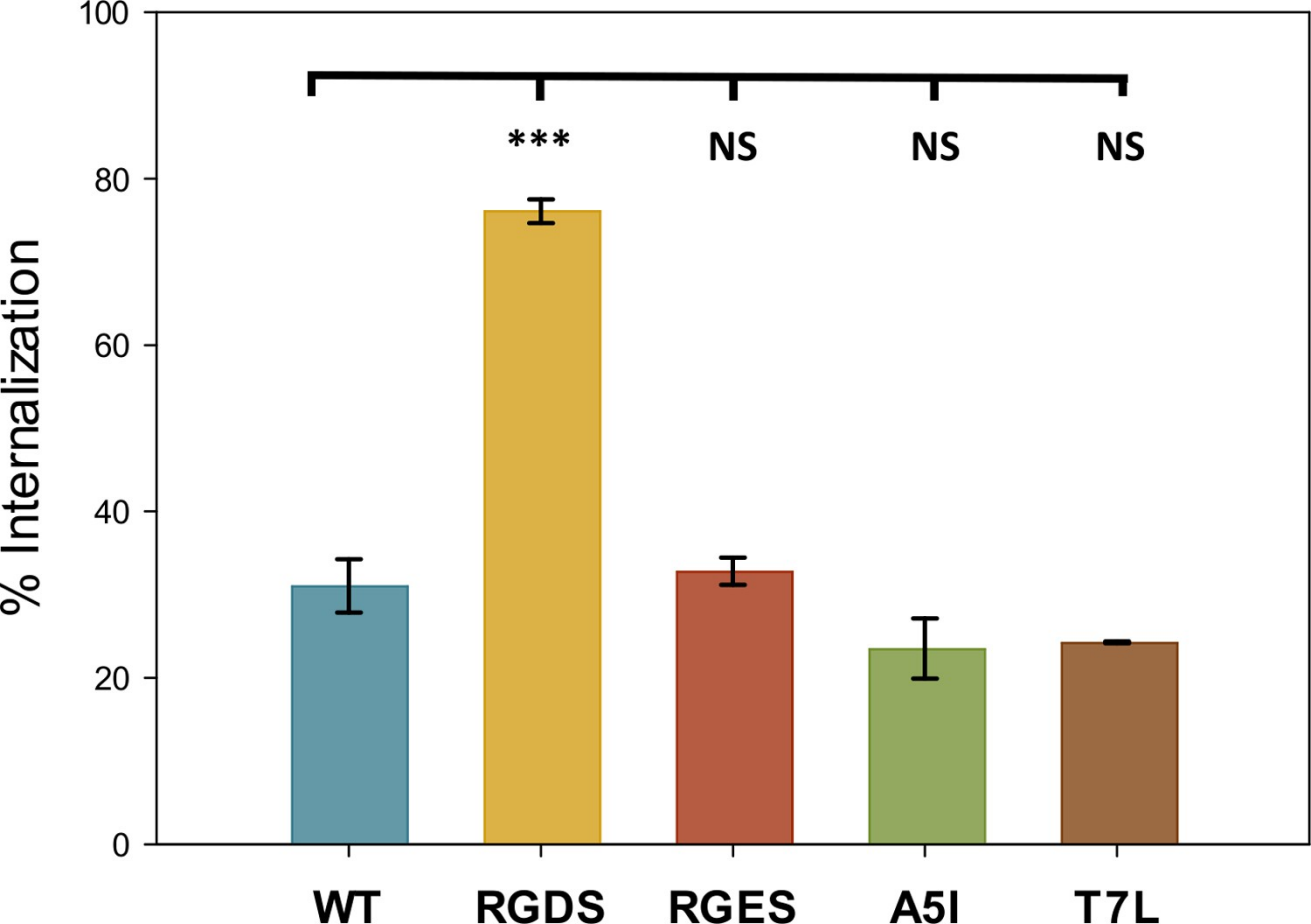
Effect of Caf1 mutant proteins on the phagocytosis of polystyrene beads. Polystyrene beads were coated with purified Caf1 polymers and incubated with J774.A1 macrophages for 2 h before fixation. Non-phagocytosed beads were visualised by incubation with a mouse anti-Caf1 antibody, followed by a goat anti-mouse Alexa Fluor 555 antibody, that fluoresces red. Phagocytosed beads were visualised following permeabilization with Triton X-100 by probing with the mouse anti-Caf1 antibody, followed by a goat anti-mouse Alexa Fluor 488 antibody, which fluoresces green. Fifty macrophages were randomly chosen to calculate internalisation, with the identity of the sample unknown to the experimenter. The percentage of internalised beads was determined by comparing the number of external (red) beads to the total number of beads (green). Error bars represent the standard deviation of three independent biological replicates. Data were analysed by one way ANOVA with a Holm-Sidak post-hoc test. NS–Not significant, ***—P < 0.005.

Beads coated with F1^WT^ were not readily phagocytosed, in contrast to the beads coated with F1^RGDS^ where the majority of the beads were engulfed (**Figs 3 and **S4). As expected, beads coated with the low affinity F1^RGES^ protein were engulfed at a similar low level to the F1^WT^ coated beads (**Figs 3 and **S4). The lower stability F1^A5I^ and F1^T7L^ coated beads were internalised at low levels similar to the Caf1^WT^. (**Figs 3 and S**4),

To provide further evidence that the lower stability mutants were not more readily recognised by macrophage receptors than the wild-type protein, a 2D cell adhesion assay was conducted, where plastic surfaces were coated with Caf1 polymers and the number of HeLa cells or macrophages that had adhered to the surface after 24 h were observed. For the HeLa cells (**S5 Fig**), large numbers of cells could be seen to adhere to the uncoated and F1^RGDS^ coated surfaces, whilst the F1^WT^, F1^RGES^, F1^A5I^ and F1^T7L^ coated surfaces supported the attachment of much fewer cells. For the macrophages (**S5 Fig**), a similar pattern was observed, although the F1^A5I^ and F1^T7L^ coated surfaces supported the attachment of an intermediate number of cells, much fewer than the uncoated and F1^RGDS^ surfaces but more than the F1^WT^ and F1^RGES^ coated surfaces. Together, these results show that, similar to F1^WT^ and unlike the F1^RGDS^, the F1^A5I^ and F1^T7L^ proteins are not easily recognised by macrophages. Therefore, their lack of protective ability observed in the bacterial phagocytosis assay must come from another property of these mutant proteins.

### Mechanical stability of Caf1 proteins

**Fig 4:**
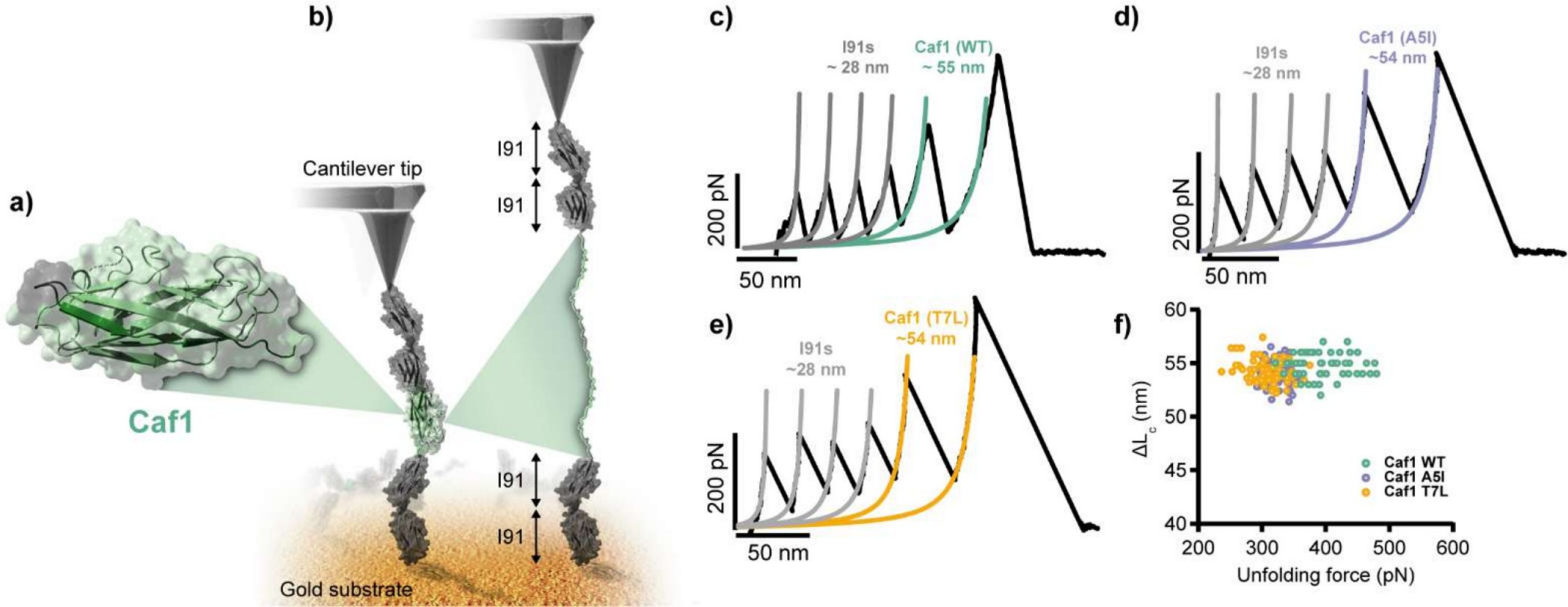
Mechanical stability of Caf1 mutants. **(a)** Close up view of a self-complemented Caf1 (cpCaf1) monomer. The gene encoding this monomer was inserted in the middle of 4x I91 protein domains from titin to produce the I91_2_-cpCaf1-I91_2_ construct. (**b**) The I91_2_-cpCaf1-I91_2_protein was attached to a gold substrate at one end and to a cantilever tip on the other. When a tensile force is applied to Caf1 it elongates until breaking point at which it is completely unfolded. (**c-e**) Force unfolding curves of I91_2_-cpCaf1^WT^-I91_2_, I91_2_-cpCaf1^A5I^-I91_2_ and I91_2_-cpCaf1^T7L^-I91_2_. Coloured lines represent fits of the worm-like chain model to the data. (**f**) Plot of extension vs. unfolding force for I91_2_-cpCaf1^WT^-I91_2_ (green), I91_2_-cpCaf1^A5I^-I91_2_ (purple) and I91_2_-cpCaf1^T7L^-I91_2_ (yellow). Individual unfolding experiment are shown as individual data points coloured according to the protein used.

As macrophages are known to exert forces on their targets during phagocytosis [4–6], and the N-terminal donor strand of Caf1 is important for polymer stability [32,33], we hypothesised that the mechanical stability of F1 might be an important property in determining its protective ability, and that our substitutions in this strand may affect this stability. Therefore, we determined the mechanostability of wild-type F1, F1^A5I^ and F1^T7L^ using single molecule force spectroscopy (SMFS), a technique we had previously used for members of another chaperone usher protein family, Fim, that form adhesive polymers on *E*. *coli* cells [31]. Briefly, we constructed a polyprotein in which a circularly permuted *caf1* subunit (cpCaf1, where the N-terminal donor strand is placed on the C-terminus after a flexible linker, completing the Ig-like fold, **Fig 4A**) was bracketed with two tandem I91 domains from the cardiac protein titin, that are used as a mechanical fingerprint on account of their well-known properties [31]. The final constructs (I91_2_-cpCaf1-I91_2_, I91_2_-cpCaf1^A5I^-I91_2_, I91_2_-cpCaf1^T7L^-I91_2_) terminate with a cysteine to facilitate adhesion to the gold surface (**Fig 4B**). The structure and thermostability of the cpCaf1 [34] and similar self-complemented subunits [32] have been characterised previously and match those of polymeric Caf1.

In the SMFS experiments a cantilever tip, with a single protein absorbed, retracts at a constant speed of 400 nm/s. With the protein captured between the tip and the gold surface, the force exerted triggers the unfolding of the protein, which can be monitored as a force-extension peak (**Fig 4C–4E**). In our polyprotein, we first observed the unfolding of the I91 domains at a typical force of around 200 pN with an increment in contour length of 28 nm. We subsequently observed a higher peak of 54–55 nm that we attribute to cpCaf1, which unfolds at a force of 394 ± 40 pN (**Fig 4C**). This value is very high compared to other proteins, which typically unfold at forces between 25–250 pN [35], but similar to other CU proteins, such as the Fim proteins, which have unfolding forces within the range of 350–530 pN [31]. In comparison, cpCaf1^A5I^ and cpCaf1^T7L^ unfold at forces of 318 ± 21 pN and 310 ± 30 pN respectively (**Fig 4D–4F**). Therefore, the point mutations caused a drop in mechanostability of approximately 20%. Together with the results of the phagocytosis assay, these data indicate that the mechanostability of Caf1 is not only essential for its anti-phagocytic capability but that its magnitude is surprisingly close to the minimal effective value.

## Discussion

Although F1 was well known to protect bacteria from phagocytosis, the specific molecular mechanism had not been defined [17]. Here, we demonstrate that the anti-phagocytic activity of F1 is dependent on three factors: its low affinity for cells in general, its lack of specific ligands for critical macrophage receptors and its high mechanical stability.

### Determinants of anti-phagocytic activity: Low binding to cell surfaces

Compared to uncoated, control *E*. *coli*, far fewer F1 coated bacteria were found associated (surface bound or engulfed) with macrophages. This agrees with the observation that F1 coated surfaces interact poorly with cells in general [17]. Surprisingly, bacteria expressing Caf1^RGDS^ showed no increase in attachment to macrophages, revealing that even a dense coverage of integrin ligands cannot reverse the fundamental non-adherent phenotype. This behaviour may be explained in simple molecular terms since purified F1 also demonstrates a notable resistance to self-aggregation *[36]* and even at high concentrations the long polymers do not gel but behave as a viscous liquid *[30]*. This is reminiscent of highly hydrated polymers such a polyethylene glycol (PEG) which are used to artificially reduce cell surface interactions. Coupled to this, Caf1 has a pI of 4.5, giving it a net negative charge at physiological pH which will repel both other F1 polymers and cell surfaces, which are generally negatively charged too. These parameters could explain the low association of any F1 coated cells to macrophages in our assays. Furthermore, in the case of extensively researched artificial polymer brushes, such as PEG, which is used to resist protein binding to implanted medical devices, there is still debate whether the effect is due to weak interactions leading to a low equilibrium binding level or a kinetic barrier which massively slows down the binding rate [37]. If the brush-like surface of Caf1 (see **Fig 2**) imposes a kinetic barrier then the specific affinity of the macrophage for bacteria coated with F1^RGDS^ is unimportant unless the bacteria reach the macrophage surface.

### Determinants of anti-phagocytic activity: Low affinity for macrophage receptors

Macrophage integrins are critical receptors which, when activated by specific ligand binding, initiate the signalling pathways which drive engulfment. Ligands of the α_M_β_2_ integrins include bacterial LPS which promotes recognition and engulfment of Gram negative bacteria such as *E*. *coli*, whilst α_5_β_1_ integrins bind the RGDS motifs of fibronectin and vitronectin [38–40] to enable macrophages to either eliminate apoptotic cells or migrate to sites of infection to increase their bactericidal activity [38,41]. The addition of RGDS to Caf1 allows the macrophages to phagocytose the few bacteria attached to their surface. This effect is specific, as it was not observed when the highly similar but inactive RGES sequence was inserted instead. Furthermore, polystyrene beads coated in F1^RGDS^ can be recognised and engulfed by macrophages, but beads coated with F1^WT^ or F1^RGES^ cannot. F1^RGDS^ coated plastic surfaces also support the adhesion of both macrophages and HeLa cells, whereas F1^WT^ and F1^RGES^ do not. In summary, these data suggest that Caf1 lacks the molecular signals that promote engulfment and, by also masking the organism’s own PAMPs (such as LPS), prevents receptor recognition, allowing bacteria to escape phagocytosis. In the future Caf1 could be used as a platform to discover other sequence motifs that play roles in engulfment.

### Determinants of anti-phagocytic activity: Mechanical strength

We show here that the F1 polymer exhibits a high mechanical stability that single amino acid substitutions in the donor strand can reduce by approximately 80–90 pN (20%). Such substitutions were known to lower the thermodynamic stability of the subunit-subunit interface, causing a drop in melting temperature [32], but the effect on mechanostability was unknown. Crucially, SDS-PAGE analysis and TEM images revealed that, despite their lower stability, the mutant proteins still form SDS-resistant polymers, which coat the bacterium. Additionally, we have shown that the F1^A5I^ polymers formed are long, as they can be purified using a 500 kDa molecular weight cut-off filter (corresponding to a minimum of 32 subunits with a length of >190 nm [33]). However, whilst the ~20% reduction in F1 mechanostability does not affect the polymer’s structure, it eliminates F1 mediated protection from phagocytosis. Polystyrene beads coated in the F1^A5I^ and F1^T7L^ were not phagocytosed at levels any higher than those coated with F1^WT^ and HeLa cells adhered to these proteins at similarly low levels to F1^WT^, indicating that these proteins are not recognised by the macrophages any more readily than the wild-type. Since the loss of its anti-phagocytic behaviour is not due to incomplete F1 polymer formation, spontaneous polymer breakdown **(Figs 1C and S1)**, increased recognition by macrophage receptors (**Fig 3)** or shedding of the Caf1 coat **(Figs 1B and** S3**)** we propose that it is related to the mechanics of the macrophage interface. The RGDS and RGES insertions made in the F1^RGDS^ and F1^RGES^ are not located at the subunit-subunit interface and have very little effect (<2°C) on the thermostability of the F1 polymer [21].

### Role of macrophage contractile forces in the anti-phagocytic mechanism of Caf1

**Fig 5:**
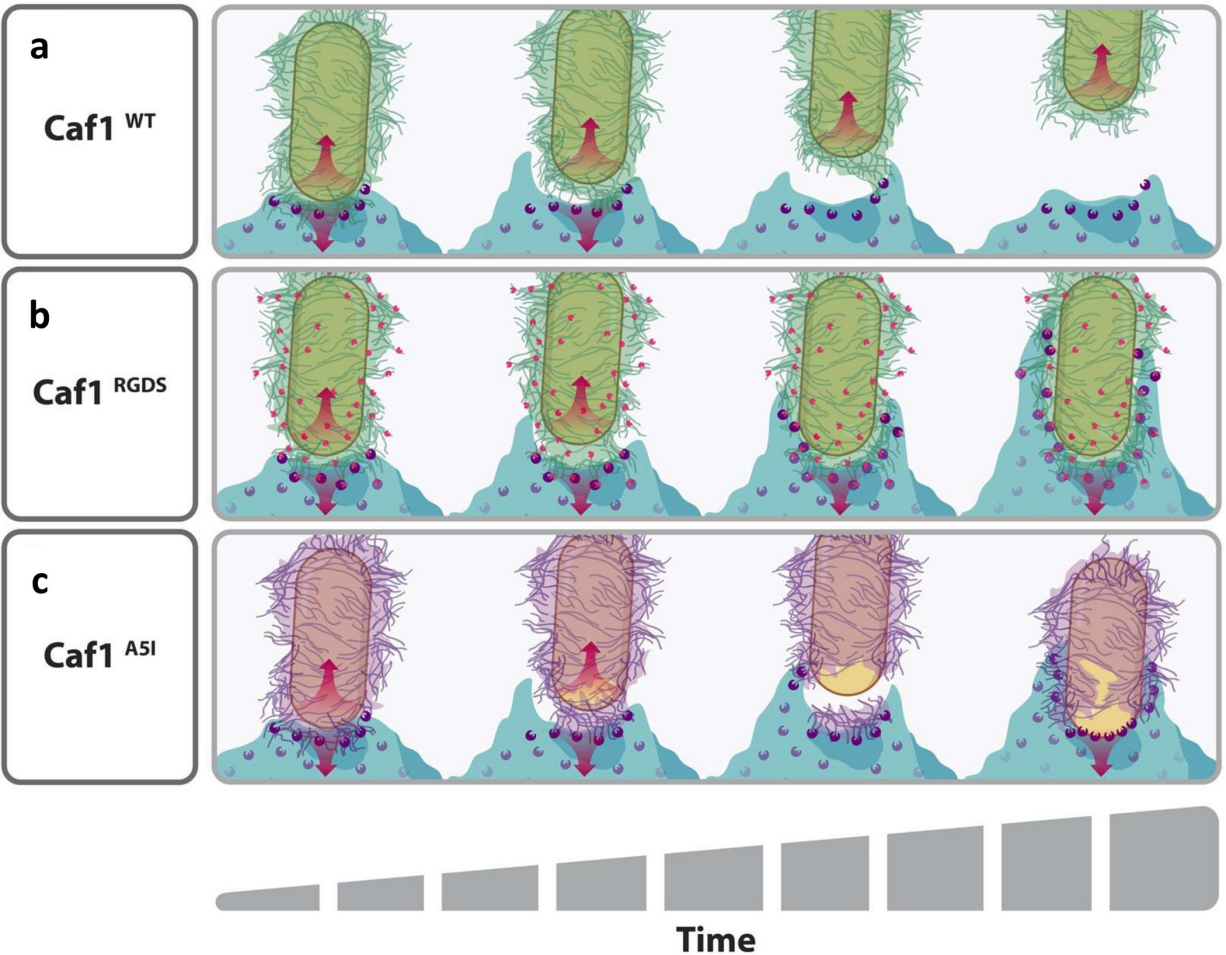
Potential mechanism for F1 mediated bacterial escape from phagocytosis. The F1 coat (green) of an encapsulated *E*. *coli* cell (tan) on the surface of a macrophage (light blue) is engaged by macrophage receptors (purple). The macrophage exerts a force on the bacteria (downwards red arrow). Brownian motion and bacterial motility provide forces in the opposing direction (red upwards arrow). (**a**) The F1 coat is mechanically strong and so does not break. The receptor- F1 interaction is weak and releases under the strain, allowing the bacterium to escape phagocytosis. In (**b**), the RGDS mutation (pink dots) is incorporated into F1. This allows the macrophage receptors to bind with higher affinity to the F1 coat, so that the bacterium can no longer escape phagocytosis and is pulled towards the macrophage, resulting in further receptor engagement and clustering, and the formation of a phagocytic cup. In (**c**), the A5I mutation reduces the mechanical strength of the F1 coat. Therefore, when the macrophage exerts a force on the coat, it experiences a strain and breaks to reveals the surface of the bacterium (tan). This is readily recognised by the macrophage and allows the bacteria to be phagocytosed.

One potential mechanism through which this process could occur is through disruption of the Caf1 coat by the macrophage [5,8,42] (**Fig 5**). This could occur when an engaged bacterium attempts to move away from a macrophage (either by Brownian motion or bacterial motility), or as receptors bound to the bacterial surface apply stretching forces on account of the increase in membrane tension which accompanies the stages after cup formation [8]. We suggest that wild-type Caf1 capsules can resist such forces, and allow bacteria to escape phagocytosis, whilst weaker capsules such as A5I could be disrupted, exposing bacterial surface PAMPs and facilitating engulfment. In favour of this hypothesis, bacteria coated with the lower stability Caf1 mutants are readily engulfed by macrophages, whereas polystyrene beads that are similarly coated and have no PAMPs, are not. Furthermore, HeLa cells do not readily adhere to surfaces coated with the lower stability Caf1 mutants, but macrophages show slightly increased levels of adhesion in these conditions compared to F1^WT^ and F1^RGES^ coated surfaces, though not as high as those found on uncoated plastic and F1^RGDS^ coated surfaces. It is possible that the macrophages can disrupt the lower stability coatings and adhere to the plastic surfaces beneath, resulting in their intermediate level of adhesion.

Previous studies measuring the forces that can be generated by macrophages have produced a range of values, from the pN to the nN scale [4,5,42–45]. We observe that a drop in F1 mechanostability from ~400 to ~320 pN is accompanied by a reversal of its anti-phagocytic activity. It is a general principle that proteins evolve a stability only marginally higher than necessary for their function [46,47], so it is possible that the stretching force exerted by the macrophage on *individual* molecules of Caf1 is within this evolved capability. Furthermore, is not inconceivable that Caf1’s low adherence and high stability have co-evolved to a point where the imparted force, can be resisted by a sufficiently strong polymer. As the macrophage likely exerts this force on several Caf1 molecules at once during attachment, the overall force exerted by the macrophage on the bacterium could be on the nN scale observed previously. Caf1 mutagenesis coupled with SFMS thus provides additional information on the mechanical stresses that occur during phagocytosis, adding a new method to probe this important area of cell mechanics [7].

## Methods

### Plasmids and cloning

pGEM-T (Promega) was used as the basis for all subsequent plasmids. Caf1 and its mutants were expressed from the pT7-COP and pT7-COPΔR plasmids [26,33], which contain T7-dependent transcriptional units comprising either the full *caf1* operon (*caf1R*, *caf1M*, *caf1A and caf1)* or the operon where *caf1R* has been deleted (*caf1M*, *caf1A*, *caf1)*, which we have observed results in higher levels of *caf1* expression [33]. F1^RGDS^, F1^RGES^, F1^A5I^ and F1^T7L^ mutants were cloned using the sequence and ligation independent cloning (SLIC) method [48] with pT7-COPΔR as a template, where primer sequences are displayed in **S1 Table.** Mutant protein sequences are shown in **S2 Table.** pT7-COPΔCaf1 and pT7-COPΔRΔCaf1 were generated through the substitution of codon 4 for a stop codon, i.e. the wild-type sequence, MKKISS, was mutated to MKK-stop, hence causing a knock-out of the gene through premature truncation of translation.

I91_2_-cpCaf1-I91_2_ was synthesised as a double stranded DNA insert by GeneArt (ThermoFisher Scientific), and ligated into a linear pQE80L plasmid by the SLIC method [48]. I91_2_-cpCaf1^A5I^-I91_2_ and I91_2_-cpCaf1^T7L^-I91_2_ were then generated from this plasmid by PCR mutagenesis using the SLIC method [48]. Primer and protein sequences are shown in **S1** and **S2 Tables** respectively.

### Protein expression

*E*. *coli* BL21(DE3) cells (NEB) were transformed with the relevant plasmid and grown in 5 mL Terrific Broth (TB) cultures supplemented with 100 μg/mL ampicillin at 35°C for 22 h in order to express Caf1 proteins. For expression of the I91_2_-cpCaf1-I91_2_ constructs, *E*. *coli* BL21(DE3) cells were transformed with pQE80L plasmids containing the relevant coding sequences. Single colonies were then used to inoculate 500 mL of lysogeny broth (LB) medium. The cultures were grown at 37°C until the OD_600_ value was ~0.6, at which time IPTG was added to the culture to a final concentration of 1 mM in order to induce protein expression. Cultures were then grown for a further 3.5 h at 37°C, before centrifugation at 4424 x g for 15 min at 4°C. Cell pellets were resuspended in phosphate buffered saline (PBS) then centrifuged at 2367 x g for 10 min at 4°C. The cell pellets were then stored at -20°C.

### Protein purification

Caf1 polymers for coating surfaces and beads were produced as described previously [30,33,49]. For proteins that were used for SMFS experiments, frozen cell pellets were resuspended in loading buffer (50 mM Tris pH 7.4, 150 mM NaCl, 20 mM imidazole) supplemented with protease inhibitors (100 μg/mL AEBSF, 100 μg/mL Benzamide, 0.5 μg/mL Aprotinin, 1 μg/mL. Pepstatin and 1 μg/mL Leupeptin). Cells were lysed using a OneShot cell disruptor operated at 20 kPSI of pressure. The lysate was then clarified by centrifugation at 43667 x g for 30 min, then 39191 x g for 20 min at 4°C. 4 mL of Nickel-NTA resin in a gravity flow column was equilibrated in loading buffer before application of the lysate to the resin. Bound proteins were washed with 3 column volumes of loading buffer before elution with a solution containing 50 mM Tris pH 7.4, 150 mM NaCl and 500 mM imidazole. Fractions were analysed for protein content by SDS-PAGE, then relevant fractions pooled and applied to a ProteoSEC S75 column (Generon), pre-equilibrated in PBS. Elution fractions were analysed by SDS-PAGE, and fractions containing the I91_2_-cpCaf1-I91_2_ proteins pooled and concentrated using Vivaspin 6 30 kDa molecular weight cut-off centrifugal concentrators (Sartorius). The concentration of the final samples was determined using UV absorbance at 280 nm and samples were flash frozen using liquid nitrogen for storage.

### Transmission electron microscopy

Cultures of transformed BL21(DE3) *E*. *coli* (NEB) were grown in TB media supplemented with antibiotic for 24 h at 35°C. Carbon coated copper electron microscopy grids were glow discharged, then incubated with 20 μL of culture for 5 min. The bacteria were then fixed by a 5 min incubation with 20 μL 2% glutaraldehyde. The grid was then washed 1–2 times with 20 μL water, before a 30 s incubation in 20 μL 2% uranyl acetate for negative staining. Grids were then visualised using a Hitachi HT7800 120kV transmission electron microscope (EM Research Services, Newcastle University). Images were recorded in tagged image file format (TIFF).

### Coating of polystyrene beads

100 μL of 1 μm diameter Fluroesbrite PolyFluor 511 polystyrene beads (Polysciences) was centrifuged at 5000 x g and resuspended in 100 μL 50 mM sodium acetate buffer, pH 5.0. F1 proteins were diluted to 0.4 mg/mL in the same buffer and added to the beads in a 1:1 ratio. The solutions were incubated on a roller for ~ 16 h at 4°C, centrifuged at 5000 x g, resuspended in the 200 μL fresh sodium acetate buffer, before centrifuging again at 5000 x g and resuspending in 200 μL fresh sodium acetate buffer. This resulted in a solution of 2.275 x 10^10^ beads/mL, and successful protein coating was determined by dot blot using a mouse anti-Caf1 antibody (Stratech) at a 1/1000 dilution and a goat anti-mouse alkaline phosphatase conjugated secondary antibody (Proteintech) also at a 1/1000 dilution.

### Phagocytosis assay

J774.A1 (mouse macrophage-like; ATCC_TIB-67) cells were seeded (~1.1x10^5^) in Dulbecco’s Modified Eagle’s Media (DMEM) supplemented with 10% fetal calf serum (FCS) on glass coverslips 2 days prior to infection to obtain 60–70% confluence on the infection day. *E*. *coli* cultures were grown overnight in Lysogeny Broth (LB) media (with antibiotic when appropriate) prior to determining the OD_600_ value and calculating the volume for a Multiplicity of Infection (MOI) of 100:1 (bacteria to J774.A1 macrophage). Thirty minutes prior to infection, the macrophages were washed (37°C PBS) and incubated in DMEM only. Once inoculated, macrophages were centrifuged for 5 min at 500 x g, 37°C and incubated for 1 h. Macrophages were then washed (37°C PBS) and incubated in DMEM containing chloramphenicol (bacterial protein synthesis inhibitor; 25 μg/ml final concentration) for 2 h to promote bacterial uptake. Finally, the cells were washed twice (ice cold PBS) and fixed by incubating 20 min with PBS containing 2.5% para-formaldehyde (PFA; Santa Cruz Biotechnology), then stored at 4°C.

The percentage of bacterial internalisation was determined as previously described [25]. Briefly, extracellular bacteria were labelled by incubating 30 min (at RT) with 1/100 rabbit anti-*E*. *coli* all serotypes antibodies (Abcam) diluted in PBS. Following three washes with PBS, the cells were incubated for 30 min with 1/100 diluted goat anti-rabbit Alexa-555 conjugated secondary antibodies (Molecular Probes). The cells were then washed three more times with PBS. All cell-associated bacteria were labelled by incubating with the same primary antibody (PBS containing 1% Triton X-100, which makes macrophage membrane permeable to antibodies) followed by goat anti-rabbit Alexa-488 conjugated secondary antibodies (Molecular Probes), at a 1/100 dilution. The dye 4’6-diamidino-2-phenylinodole (DAPI—fluoresces blue) was routinely added in final antibody incubations to detect bacterial and host DNA. Coverslips were placed onto FluorSave reagent (Calbiochem) on glass slides (Thermo Scientific) for phase contrast/fluorescent microscopy examination (Zeiss Axioskop Epifluorescence microscope).

Fifty macrophages were randomly selected to count the number of extracellular and total-cell associated bacteria enabling the percentage internalisation to be calculated. These studies were undertaken in a semi-blind manner i.e. samples were placed in a withheld order until all counting was complete, and the percentage of internalisation calculated.

Phagocytosis assays involving polystyrene beads were performed similarly, with the following differences. Cells were incubated for 2 h with 10 μL of the coated polystyrene beads, before fixation. Extracellular beads were labelled by incubating 30 min at room temperature with a mouse anti-Caf1 antibody (Gene Tex) diluted 1/50 in PBS. Following three washes (PBS), the cells were incubated 30 min with 1/100 goat anti-mouse Alexa-555 conjugated secondary antibodies (Molecular Probes). The cells were then washed again (3 x with PBS) with all cell-associated beads labelled by incubating with the same primary antibody diluted 1/50 in PBS containing 1% Triton X-100 to permeabilise the cells. This treatment was followed by 1/100 goat anti-mouse Alexa-488 conjugated secondary antibodies (Molecular Probes). Percentage internalisation of the beads was then calculated in the same way as for the bacteria.

### Adhesion assay

1 mL solutions of 1 mg/mL of F1^WT^, F1^RGDS^, F1^RGES^, F1^A5I^ and F1^T7L^ proteins in water were added to wells of a 24-well plastic plate (Corning), which was then incubated at -80°C for 1 h, before freeze drying for 24 h. 1 mL of DMEM containing 10% FCS, Penicillin-Streptomycin (Sigma-Aldrich) and 1 x 10^5^ J774.A1 (mouse macrophage-like; ATCC_TIB-67) or Hela cells (ATCC-CCL-2) were added to the coated wells, as well as an uncoated well, and then incubated for 24 h at 37°C. Pictures were taken using an EVOS bright-field imaging system (Thermo Fisher).

### Single molecule force spectroscopy

*Single-molecule force spectroscopy experiments were carried out using a commercial atomic force spectroscope (Luigs and Neumann)*. The cantilevers used in the experiments were calibrated using the equipartition theorem and they had typical spring constant of around 6 pN/nm (Bruker OBL-10). Proteins were incubated for ten minutes over custom made gold surfaces at a concentration of 0.1–1.0 g/L. The buffer used was HEPES 10 mM pH 7.0, NaCl 150 mM and 1 mM EDTA. Force-extension experiments were performed at 400 nm/s. The traces obtained were collected and analyzed with a custom-written code using the worm-like chain model for polymer elasticity. All the figures were generated using Igor Pro (Wavemetrics) and Adobe Illustrator (Adobe).

## Supporting information

S1 Fig Caf1 polymer formation by transformed *E*. *coli* cells. (DOCX)

S2 Fig Representative images of macrophages challenged with *E*. *coli*. (DOCX)

S3 Fig Transmission electron micrographs of *E*. *coli* expressing *caf1* mutants. (DOCX)

S4 Fig Representative images of macrophages challenged with F1 coated polystyrene beads. (DOCX)

S5 Fig 2D cell adhesion assay with F1 coated surfaces. (DOCX)

S1 Table DNA Primer sequences used in this study. (DOCX)

S2 Table Amino acid sequences of proteins generated for this study. (DOCX)
