# Supplementary material for: Unraveling the molecular determinants of the anti-phagocytic protein cloak of plague bacteria
Source: PLoS Pathog. 2022 Mar 31;18(3):e1010447. doi: 10.1371/journal.ppat.1010447 (PMC9004762; doi:10.1371/journal.ppat.1010447)
Supplement: S1 Fig — (DOCX) [file ppat.1010447.s001.docx]

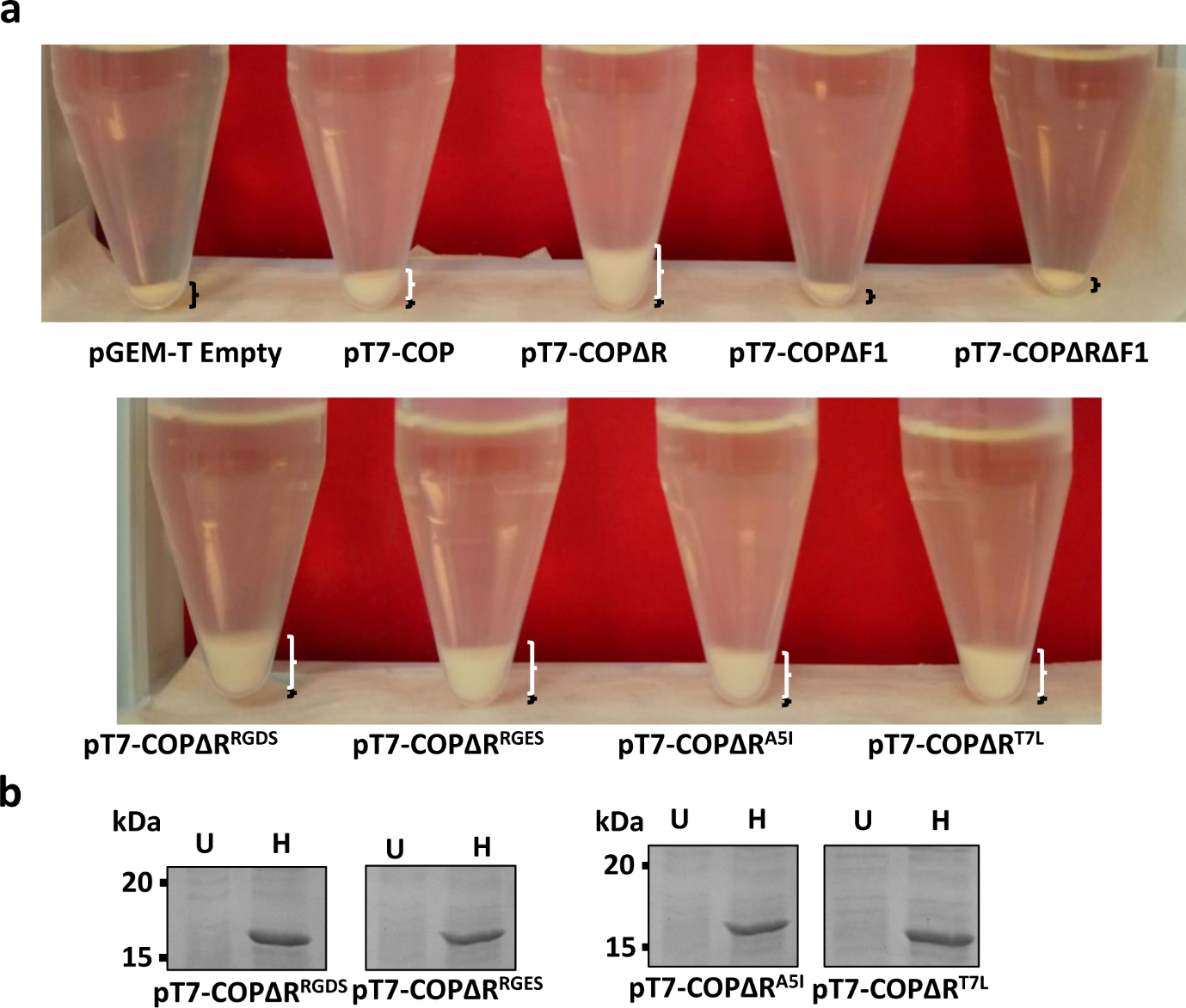


**S1 Fig: Caf1 polymer formation by transformed *E. coli* cells.** (**a**) *E. coli* cells were transformed with the indicated plasmids and grown for 22 h at 35°C to induce *caf1* expression. After growth, 2 mL of culture was centrifuged at ~2300 x g for 15 minutes and examined for flocculent layer production. Pellet heights are shown using black boundaries, whilst flocculent heights are shown with white boundaries. In cases where the flocculent production was high, pellet sizes were very small, and the boundaries shown here are approximations. (b) SDS-PAGE analysis of the extracellular fraction (comprising flocculent and supernatant) of cultures of *E. coli* transformed with the indicated plasmids and grown for 22 h at 35°C. Samples were incubated at either room temperature (unheated, U) or 100°C (heated, H) for 5 minutes prior to loading on the gel.
