# Supplementary material for: Unraveling the molecular determinants of the anti-phagocytic protein cloak of plague bacteria
Source: PLoS Pathog. 2022 Mar 31;18(3):e1010447. doi: 10.1371/journal.ppat.1010447 (PMC9004762; doi:10.1371/journal.ppat.1010447)
Supplement: S2 Fig — (DOCX) [file ppat.1010447.s002.docx]

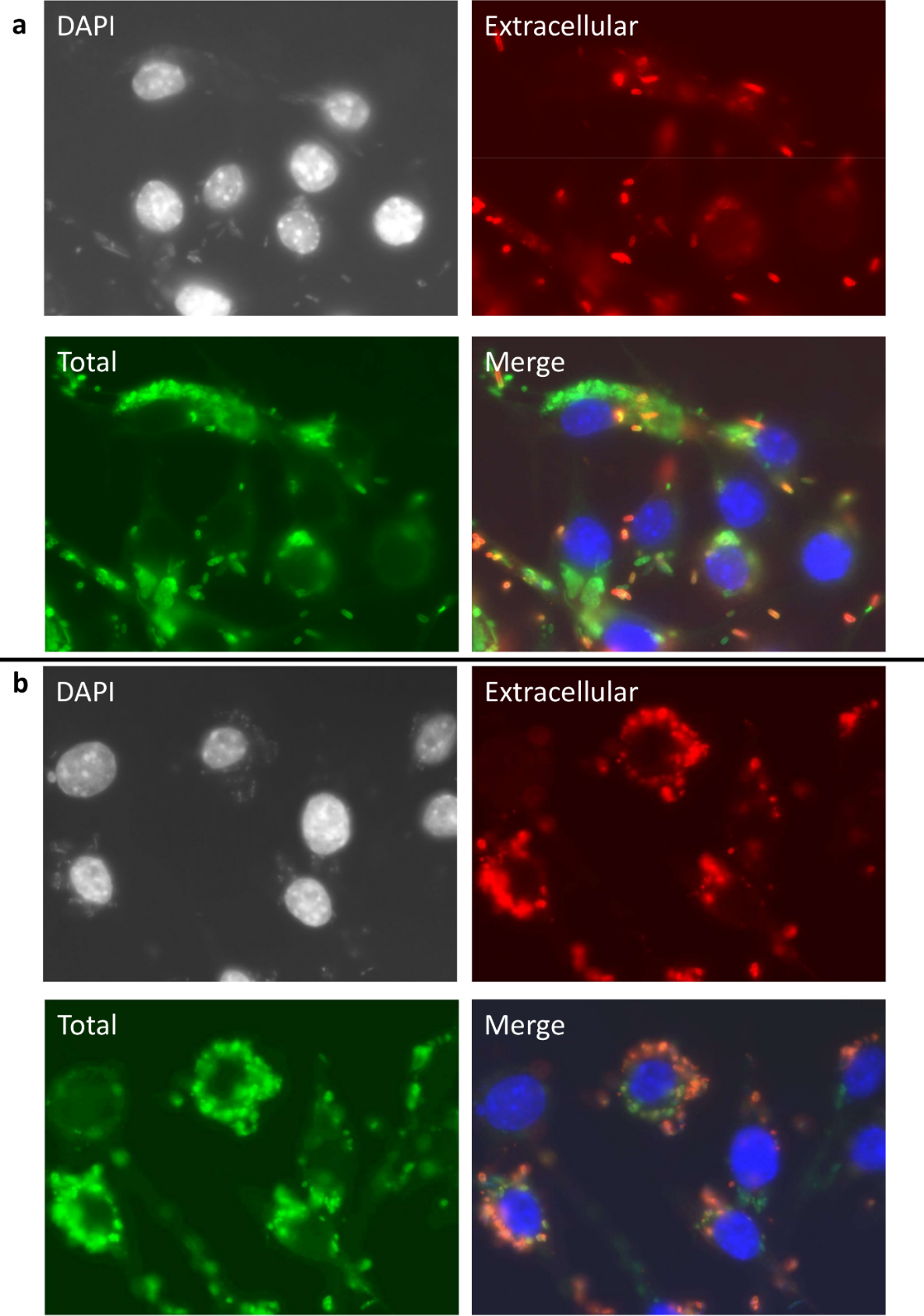


**S2 Fig: Representative images of macrophages challenged with *E. coli***. J774.A1 macrophages were infected for 2hrs with *E. coli* cells transformed with either the pT7-COPΔRΔF1 (**a**) or the pT7-COPΔR plasmid (**b**). Cells were fixed and stained with DAPI, and bacteria labelled with Alexa Fluor 555 (red, extracellular bacteria only) and Alexa Fluor 488 (green, total bacteria) antibodies. Fifty macrophages with 10-20 bacteria associated were chosen to calculate internalisation, with the identity of the sample unknown to the experimenter. The percentage of bacteria internalised by the macrophage was calculated by determining the ratio of green and red bacteria. Images were taken with a Zeiss Axioskop Epifluorescence microscope with a 100x oil objective.
