# Supplementary material for: Unraveling the molecular determinants of the anti-phagocytic protein cloak of plague bacteria
Source: PLoS Pathog. 2022 Mar 31;18(3):e1010447. doi: 10.1371/journal.ppat.1010447 (PMC9004762; doi:10.1371/journal.ppat.1010447)
Supplement: S3 Fig — (DOCX) [file ppat.1010447.s003.docx]

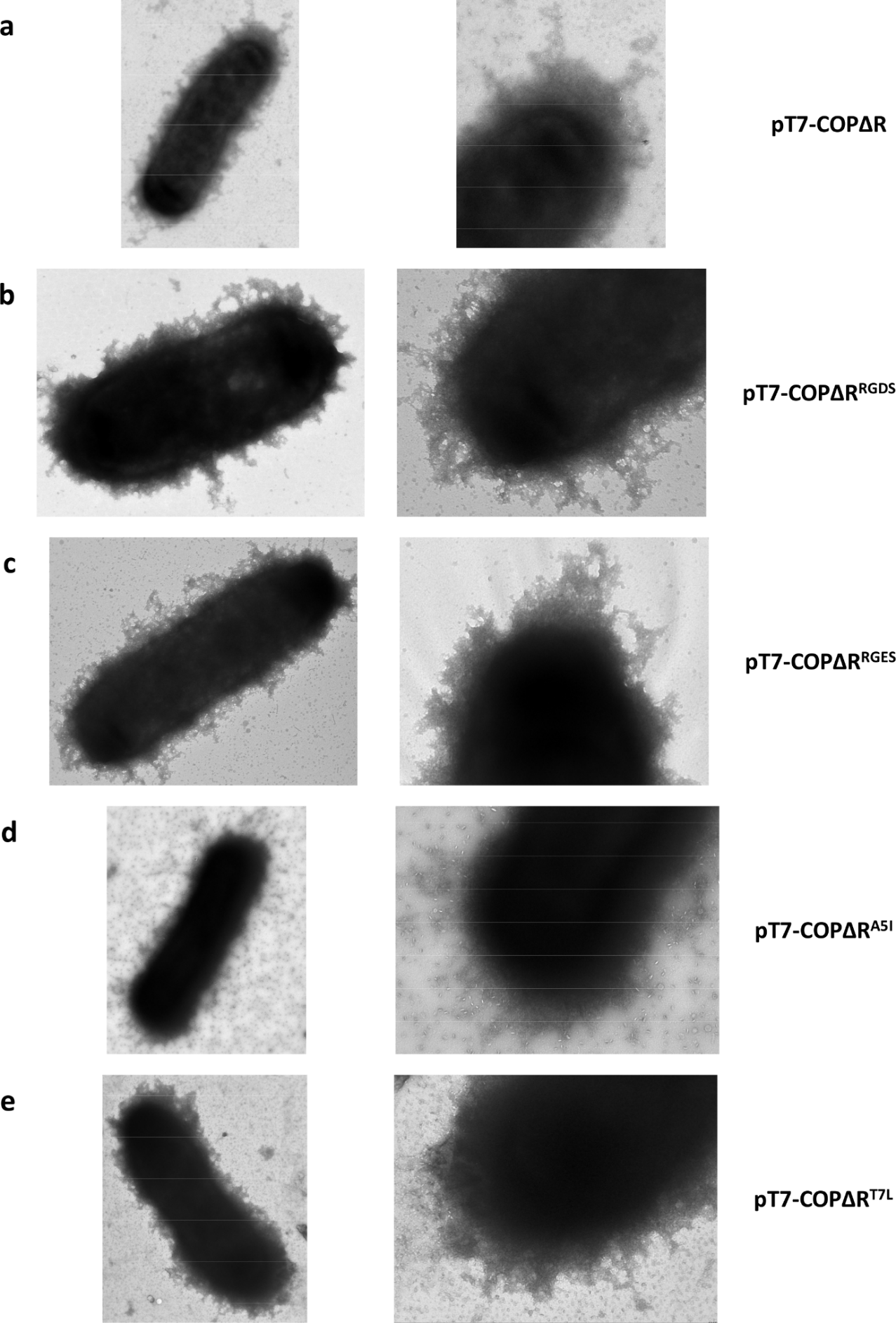


**S3 Fig: Transmission electron micrographs of *E. coli* expressing *caf1* mutants.** Close-up images of bacteria transformed with (**a**) pT7-COPΔR, (**b**) pT7-COPΔR^RGDS^ (c) pT7-COPΔR^RGES^, (d) pT7-COPΔR^A5I^ and (e) pT7-COPΔR^T7L^ are shown.
