# Supplementary material for: Unraveling the molecular determinants of the anti-phagocytic protein cloak of plague bacteria
Source: PLoS Pathog. 2022 Mar 31;18(3):e1010447. doi: 10.1371/journal.ppat.1010447 (PMC9004762; doi:10.1371/journal.ppat.1010447)
Supplement: S4 Fig — (DOCX) [file ppat.1010447.s004.docx]

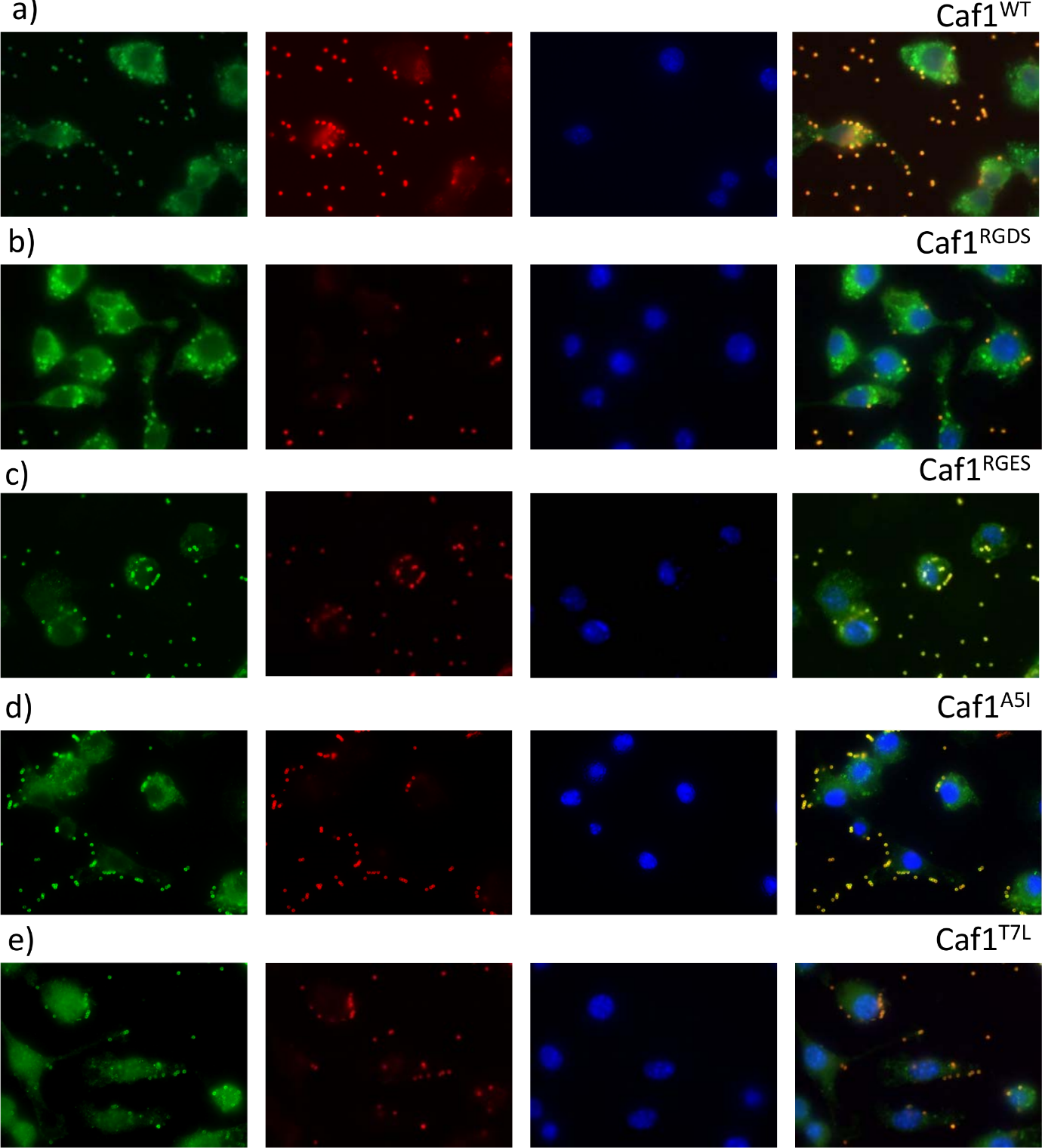


**S4 Fig: Representative images of macrophages challenged with F1 coated polystyrene beads**. J774.A1 macrophages were incubated for 2hrs with 1 µm diameter polystyrene beads coated with F1^WT^ (**a**), F1^RGDS^ (**b**) F1^RGES^ (**c**) F1^A5I^ (**d**) or F1^T7L^ (**e**). Cells were fixed and stained with DAPI, and beads visualised by incubation with a mouse anti-Caf1 antibody followed by goat anti-mouse Alexa Fluor 555 (red, extracellular beads only) and goat anti-mouse Alexa Fluor 488 (green, total beads) antibodies. The percentage of beads internalised by the macrophage was calculated by determining the ratio of green and red beads. Images were taken with a Zeiss Axioskop Epifluorescence microscope with a 100x oil objective.
