# Supplementary material for: Unraveling the molecular determinants of the anti-phagocytic protein cloak of plague bacteria
Source: PLoS Pathog. 2022 Mar 31;18(3):e1010447. doi: 10.1371/journal.ppat.1010447 (PMC9004762; doi:10.1371/journal.ppat.1010447)
Supplement: S5 Fig — (DOCX) [file ppat.1010447.s005.docx]

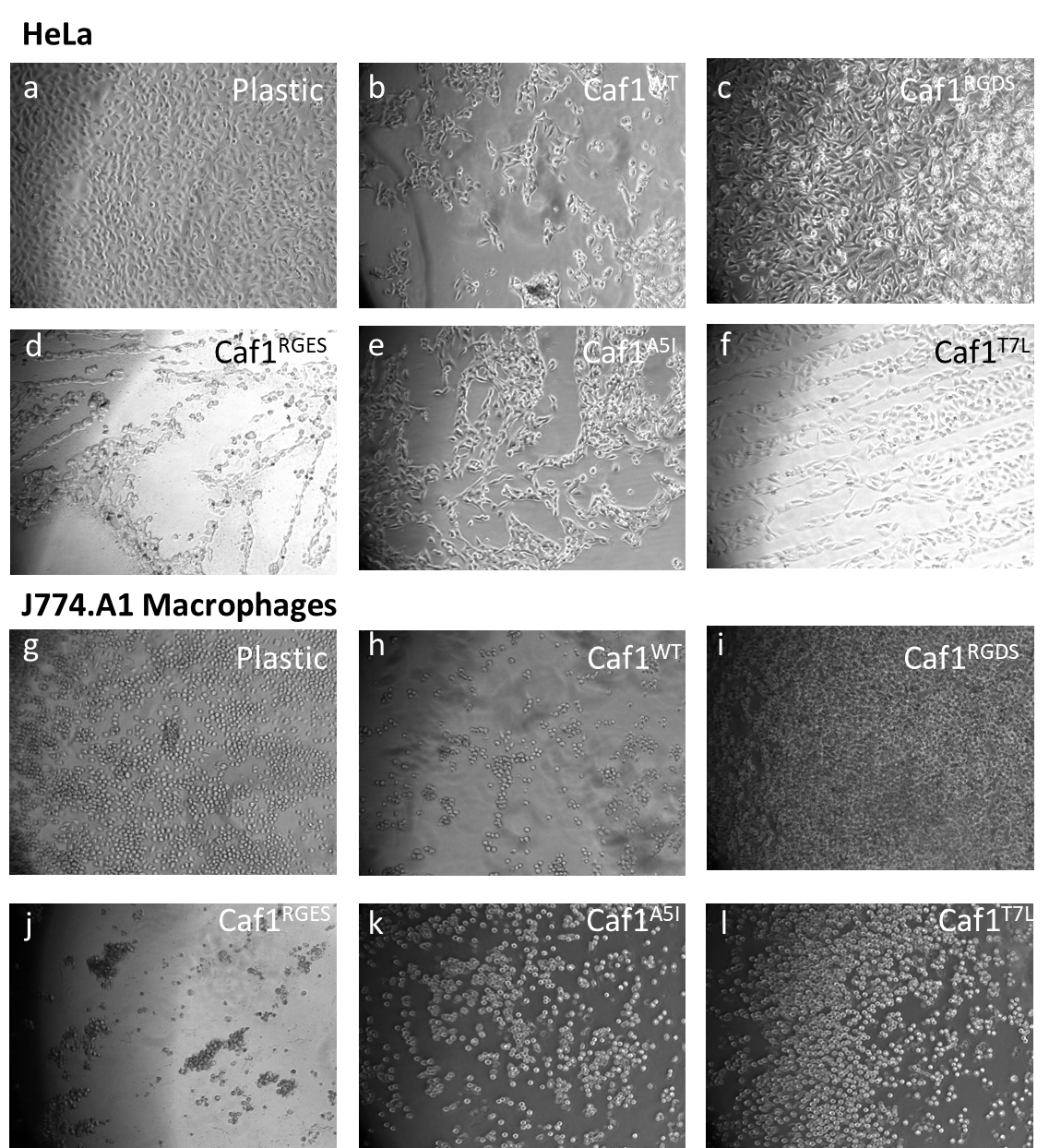


**S5 Fig: 2D cell adhesion assay with F1 coated surfaces.** 1 mL solutions of 1 mg/mL F1 protein in water was freeze dried onto the surface of a 24-well plate (with one well left uncoated) and resuspended in 1 mL DMEM supplemented with 10% fetal calf serum and penicillin-streptomycin. Wells were then seeded with either HeLa cells (**a-f**) or J774.A1 macrophages (**g-l**), which were grown at 37˚C for 24 h. Images were captured using an EVOS bright-field imaging system.
