# Supplementary material for: Unraveling the molecular determinants of the anti-phagocytic protein cloak of plague bacteria
Source: PLoS Pathog. 2022 Mar 31;18(3):e1010447. doi: 10.1371/journal.ppat.1010447 (PMC9004762; doi:10.1371/journal.ppat.1010447)
Supplement: S1 Table — (DOCX) [file ppat.1010447.s006.docx]

| **Primer** | **Sequence (5’-3’)** |
| --- | --- |
| pT7-COP Caf1R Deletion Forward | taatcctaatgttacagaatataacccaaatcaaaataatag |
| pT7-COP Caf1R Deletion Reverse | gtaacattaggattaccaaagag |
| pT7-COP^RGDS^ Forward | gtcgtggtgatagcggtggtgacgtcgtcttggctacg |
| pT7-COP^RGDS^ Reverse | cgctatcaccacgaccaccgttctcaccgtttaccttagg |
| pT7-COP^RGES^ Forward | gtcgtggtgagagcggtggtgacgtcgtcttggctacg |
| pT7-COP^RGES^ Reverse | cgctctcaccacgaccaccgttctcaccgtttaccttagg |
| pT7-COP^A5I^ Forward | agcaccactgcaacggcaac |
| pT7-COP^A5I^ Reverse | cgttgcagtggtgctaatagttaaatctgccgcattagcag |
| pT7-COP^T7L^ Forward | actgcaacggcaactcttgttg |
| pT7-COP^T7L^ Reverse | agttgccgttgcagtcaggcttgcagttaaatctgccgc |
| pT7-COP ΔF1 Forward | gtaatatatgaaaaaataaagttccgttatcgcc |
| pT7-COP ΔF1 Reverse | ggcgataacggaactttattttttcatatattac |
| pQE80L Forward | ggtaccccgggtcgacc |
| pQE80L Reverse | ggatccgtgatggtgatggtg |
| I91_2_-cpCaf1^A5I^-I91_2_Forward | agcaccaccgcaaccgcaac |
| I91_2_-cpCaf1^A5I^-I91_2_Reverse | ggttgcggtggtgctaatggtcagatctgcaccattaccg |
| I91_2_-cpCaf1^T7L^-I91_2_Forward | accgcaaccgcaaccctgg |
| I91_2_-cpCaf1^T7L^-I91_2_Reverse | ggttgcggttgcggtcaggcttgcggtcagatctgcac |

**S1 Table: DNA Primer sequences used in this study.**
