# Supplementary material for: Unraveling the molecular determinants of the anti-phagocytic protein cloak of plague bacteria
Source: PLoS Pathog. 2022 Mar 31;18(3):e1010447. doi: 10.1371/journal.ppat.1010447 (PMC9004762; doi:10.1371/journal.ppat.1010447)
Supplement: S2 Table — (DOCX) [file ppat.1010447.s007.docx]

| **Mutant Protein** | **Mutation Location** | **Mutant Sequence** |
| --- | --- | --- |
| Caf1^L5RGDS^ | Loop 5 | VNGEN*GGRGDSGG*DVV |
| Caf1^L5RGES^ | Loop 5 | VNGEN*GGRGESGG*DVV |
| Caf1^A5I^ | N-terminus | ADLT*I*STTATATLVEP |
| Caf^T7L^ | N-terminus | ADLTAS*L*TATATLVEP |
| I91_2_-cpCaf1-I91_2_ | N/A | MRGSHHHHHHGSLIEVEKPLYGVEVFVGETAHFEIELSEPDVHGQWKLKGQPLAASPDCEIIEDGKKHILILHNCQLGMTGEVSFQAANTKSAANLKVKELRSLIEVEKPLYGVEVFVGETAHFEIELSEPDVHGQWKLKGQPLAASPDCEIIEDGKKHILILHNCQLGMTGEVSFQAANTKSAANLKVKELRSPARITLTYKEGAPITIMDNGNIDTELLVGTLTLGGYKTGTTSTSVNFTDAAGDPMYLTFTSQDGNNHQFTTKVIGKDSRDFDISPKVNGENLVGDDVVLATGSQDFFVRSIGSKGGKLAAGKYTDAVTVTVSTGSGNGADLTASTTATATLVERSLIEVEKPLYGVEVFVGETAHFEIELSEPDVHGQWKLKGQPLAASPDCEIIEDGKKHILILHNCQLGMTGEVSFQAANTKSAANLKVKELRSLIEVEKPLYGVEVFVGETAHFEIELSEPDVHGQWKLKGQPLAASPDCEIIEDGKKHILILHNCQLGMTGEVSFQAANTKSAANLKVKELCC |
| I91_2_-cpCaf1^A5I^-I91_2_ | N/A | MRGSHHHHHHGSLIEVEKPLYGVEVFVGETAHFEIELSEPDVHGQWKLKGQPLAASPDCEIIEDGKKHILILHNCQLGMTGEVSFQAANTKSAANLKVKELRSLIEVEKPLYGVEVFVGETAHFEIELSEPDVHGQWKLKGQPLAASPDCEIIEDGKKHILILHNCQLGMTGEVSFQAANTKSAANLKVKELRSPARITLTYKEGAPITIMDNGNIDTELLVGTLTLGGYKTGTTSTSVNFTDAAGDPMYLTFTSQDGNNHQFTTKVIGKDSRDFDISPKVNGENLVGDDVVLATGSQDFFVRSIGSKGGKLAAGKYTDAVTVTVSTGSGNGADLT*A*STTATATLVERSLIEVEKPLYGVEVFVGETAHFEIELSEPDVHGQWKLKGQPLAASPDCEIIEDGKKHILILHNCQLGMTGEVSFQAANTKSAANLKVKELRSLIEVEKPLYGVEVFVGETAHFEIELSEPDVHGQWKLKGQPLAASPDCEIIEDGKKHILILHNCQLGMTGEVSFQAANTKSAANLKVKELCC |
| I91_2_-cpCaf1^T7L^-I91_2_ | N/A | MRGSHHHHHHGSLIEVEKPLYGVEVFVGETAHFEIELSEPDVHGQWKLKGQPLAASPDCEIIEDGKKHILILHNCQLGMTGEVSFQAANTKSAANLKVKELRSLIEVEKPLYGVEVFVGETAHFEIELSEPDVHGQWKLKGQPLAASPDCEIIEDGKKHILILHNCQLGMTGEVSFQAANTKSAANLKVKELRSPARITLTYKEGAPITIMDNGNIDTELLVGTLTLGGYKTGTTSTSVNFTDAAGDPMYLTFTSQDGNNHQFTTKVIGKDSRDFDISPKVNGENLVGDDVVLATGSQDFFVRSIGSKGGKLAAGKYTDAVTVTVSTGSGNGADLTIS*T*TATATLVERSLIEVEKPLYGVEVFVGETAHFEIELSEPDVHGQWKLKGQPLAASPDCEIIEDGKKHILILHNCQLGMTGEVSFQAANTKSAANLKVKELRSLIEVEKPLYGVEVFVGETAHFEIELSEPDVHGQWKLKGQPLAASPDCEIIEDGKKHILILHNCQLGMTGEVSFQAANTKSAANLKVKELCC |

**S2 Table: Amino acid sequences of proteins generated for this study.** Mutations are highlighted red and in italics. cpCaf1 is highlighted in blue and I91 domains in green.
